# Enzymatic Degradation of Ochratoxin A: The Role of Ultra-Pure Water

**DOI:** 10.3390/foods14030397

**Published:** 2025-01-25

**Authors:** Joana Santos, Constança Oliveira, Filipe Teixeira, Armando Venâncio, Carla Silva

**Affiliations:** 1 Centre of Biological Engineering, University of Minho, Campus de Gualtar, 4710-057 Braga, Portugal; joanasantos587@hotmail.com (J.S.); a100382@alunos.uminho.pt (C.O.); avenan@deb.uminho.pt (A.V.); 2 Centre of Chemistry, University of Minho, Campus of Gualtar, 4710-057 Braga, Portugal; fteixeira@quimica.uminho.pt; 3 LABBELS—Associate Laboratory, 4710-057 Braga, Portugal

**Keywords:** ultrapure water, enzymatic degradation, ochratoxin A, lipase, food detoxification

## Abstract

Ochratoxin A (OTA) is a toxic mycotoxin, making its removal from food essential for public health. This study examines OTA degradation by porcine pancreatic lipase (PPL) in ultra-pure water versus buffer systems through in vitro assays and molecular modeling. The results show that PPL fully degrades OTA in ultra-pure water within 7 h at 44 °C, whereas only partial degradation occurs in phosphate buffer. After 4 h, PPL in water degrades 91% of OTA, compared to only 12% in buffer. The enzyme’s half-life is longer in water (~4 h 4 min) than in phosphate buffer (~2 h 30 min), suggesting better stability in water. Other buffers, including acetate, citrate, and borate, confirmed higher degradation efficiency in low-conductivity, acidic environments similar to ultra-pure water. Additionally, using the model compound *p*-nitrophenyl octanoate (*p*-NPO), it was found that *p*-NPO degrades faster in buffer, likely due to a salting-out effect. Molecular modeling and circular dichroism analysis indicate that PPL’s secondary structure in water promotes an ideal conformation for OTA binding. This study suggests ultra-pure water as a greener, sustainable option for reducing mycotoxins in food, with broad industrial applications.

## 1. Introduction

Mycotoxins are toxic secondary metabolites produced by fungi that, when ingested, inhaled, or absorbed through the skin, can cause acute and chronic health effects in humans and animals [1,2]. The global presence of mycotoxins in food and feed has a significant impact on everyone’s health, resulting in billions of dollars in annual economic losses [3]. The mycotoxins of most concern include aflatoxins, ochratoxins, zearalenone, fumonisins, citrinin, patulin, nivalenol, deoxynivalenol, and ergot alkaloids [1].

Ochratoxins are highly toxic mycotoxins, with ochratoxin A (OTA) being the most toxic and commonly found member of the ochratoxin family. OTA is one of the top five mycotoxins that are highly regulated by the law due to its nephrotoxic, genotoxic, cytotoxic, teratogenic, mutagenic, and immunotoxic effects [4]. Furthermore, the International Agency for Research on Cancer (IARC) categorizes OTA as Group 2B, indicating that it is possibly carcinogenic to humans [1].

Focusing on control strategies to mitigate OTA contamination in food is crucial. It has only been in the last six decades, following the identification of aflatoxins, that the importance of mitigating mycotoxins for the well-being of humans and animals has been recognized [5,6,7,8,9]. Biological transformation is regarded as the most promising but challenging method to decrease the accumulation of mycotoxins, as pre- and post-harvest strategies involving chemical or physical removal are not sufficiently effective [3,10,11,12].

The degradation of OTA by isolated or crude enzymes is an effective method for detoxifying food [11,13,14,15,16,17,18]. The use of isolated enzymes is a highly efficient approach for specifically targeting OTA. This method is environmentally friendly and does not involve the use of harmful chemicals. Hydrolases, more specifically lipases, are the enzymes most used in this process of mitigation [10]. Lipases [EC 3.1.1.3] are a subclass of esterases, isolated from various species as microorganisms, plants, and animal tissues, that can catalyze a variety of reactions including hydrolysis, alcoholysis, acidolysis, transesterification, and esterification.

In biotransformation reactions, porcine pancreatic lipase (PPL) is an effective enzyme due to its cost effectiveness compared to other commercially available microbial and animal lipases [19]. PPL is a protein of 50 kDa with 448 amino acid residues. In 1996, Hermoso et al. resolved the crystalline structure of the PPL–colipase complex in the open state (PDB: 1ETH) [20]. The PPL protein consists of two distinct domains, an α/β-fold N-terminal domain (residues: 1–336), termed domain 1, and a β-sandwich C-terminal domain (residues: 337–448) referred to as domain 2. The residues Ser153, Asp177, and His264 constitute the active site of the enzyme, which is shielded by a lid region extending from Cys238 to Cys262. The activity of the enzyme is modulated by conformational changes in the vicinity of the lid [21].

In biphasic system interfaces, the lid opens due to conformational changes, producing the “open structure” of the lipases; this process is known as interfacial activation. In an open conformation, substrates can access the active site and undergo conversion. However, when the lid closes, the lipase becomes inactive by shielding the active site from the environment, thus preventing substrate access [22]. In pure aqueous environments, the lid remains predominantly closed, whereas in the presence of a hydrophobic layer, it stays open.

Hanque and Prabhu [23] proposed that the PPL in its closed conformation is stable in water, whereas the open conformation is significantly less stable and tends to transition towards the closed state. The authors also reported that despite significant fluctuations in the closed conformation of PPL, that have been observed in the presence of water, it was not possible to induce the opening movement of the lid. In the open conformation, the fluctuations are smaller, and the lid moves toward the closed conformational state, as if the structure were freezing in a more polar environment. Therefore, the reaction environment must be carefully considered when using lipases. Regarding the enzymatic degradation of OTA, most studies present degradation in phosphate-buffered solutions [13], or in culture media [14,24,25,26]; however, these studies did not report on the exploration of the solvent effect on enzymatic activity.

Santos et al. have previously demonstrated that PPL has the ability to degrade 43% of OTA and completely degrade its non-chlorinated derivative, ochratoxin B, in 9 h, using phosphate buffer (100 mM, pH 7.5) as reaction medium [13]. Abrunhosa et al. had previously performed OTA degradation using pancreatin enzyme and protease under the same buffered conditions [11].

The dietary intake of phosphate and the level of phosphate in the bloodstream are significant concerns for both individuals with kidney disease and the general population. Recent research indicates that phosphate additives in food can adversely affect individuals with normal kidney function. Over the past decade, hyperphosphatemia has emerged as a strong predictor of mortality in advanced chronic kidney disease [27]. In 2009, a groundbreaking study first established a connection between phosphate concentrations and cardiovascular disease in the general population [28]. Since then, mounting evidence has reinforced this link, showing that elevated serum phosphate levels, even if they remain within the normal range, correlate with adverse cardiovascular outcomes, including coronary artery calcification, congestive heart failure, and mortality [29]. The presence of phosphate additives in food is a significant concern, and their potential impact on health may be more substantial than previously estimated. Considering this, the optimal solution for degrading mycotoxins may be the use of the greenest solvent—ultra-pure water. Water is non-toxic to both health and the environment, and its abundant, renewable availability makes it an economically and ecologically viable choice [27]. The physical properties of water, and their changes with temperature and pressure, enable a wider range of reactions in this polar solvent than initially expected [28].

Given that the major food crops worldwide are prone to mycotoxins, it is imperative to develop safer methods for eliminating these toxins from the food supply. In recent years, there has been significant progress in using green solvents for mycotoxin degradation, as they can be used directly in food without compromising its quality.

To our knowledge, no studies have yet been published on the use of ultra-pure water for OTA degradation. Despite evidence of low PPL activity in water, this study aims to clarify, through in vitro assays and molecular modeling, how ultra-pure water enhances OTA degradation compared to buffer solutions. This potentially groundbreaking approach offers a simple yet innovative method with numerous future applications in food processing. Although PPL can be contaminated by traces of trypsin, which imparts a bitter taste, and other impurities like animal hormones, the enzyme remains highly promising [30].

## 2. Materials and Methods

### 2.1. Materials

Lipase from porcine pancreas (Type II, ≥125 U/mg) (PPL), ochratoxin A (OTA), ochratoxin alpha (OTα), di-potassium hydrogen orthophosphate, and potassium dihydrogen orthophosphate were purchased from Sigma-Aldrich (Porto, Portugal). Acetonitrile (HPLC grade), acetic acid, and syringe filters PTFE membrane were purchased from Thermo Scientific (Porto, Portugal). The ultrapure water was obtained from a Water 75 filter with a pH of 5.6 and conductivity of 0.001 mS/cm. The microplate reader used to evaluate the enzymatic activity was the Synergy H1 Multi-Mode Reader from BioTek (Shoreline, WA, USA). The LC-MS is a Thermo Scientific™ Vanquish™ Flex UHPLC system coupled to a Thermo Scientific™ Orbitrap Exploris™ 120 high-resolution accurate mass spectrometer (Thermo Fisher Scientific, Bremen, Germany). The instrument control and data processing were carried out by Xcalibur 4.5 software (Thermo Scientific).

### 2.2. Enzyme Activity

The specific enzyme activity of lipases was determined using *p*-nitrophenyl octanoate (*p*-NPO) as substrate. The standard assay was performed at 37 °C in a final volume of 4 mL containing the substrate (6 mM), the enzyme, and the assay buffer (K_2_HPO_4_ buffer, pH 7.8, 50 mM). The reaction was initiated by the addition of the enzyme and stopped with the addition of acetone. The hydrolysis of *p*-NPO was observed by measuring the formation of *p*-nitrophenol using two methods. The formation of the product was measured at a wavelength of 400 nm using spectrophotometry [31]. The product was also quantified using LC-MS according to the method presented below. One unit of enzyme activity is defined as the amount of enzyme that catalyzes the production of 1 μmol *p*-nitrophenol from the initial substrate per minute.

### 2.3. Enzymatic Degradation of Ochratoxin A

OTA degradation assays were conducted using lipase from porcine pancreas in the presence of pure water and different buffers (phosphate buffer 100 mM, pH 7.5; phosphate buffer 50 mM, pH 7.5; borate buffer 100 mM, pH 7.5; acetate buffer 100 mM, pH 5; citrate buffer 100 mM, pH 6). The procedure involved incubating the enzyme (10 mg/mL) in 1 mL of solvent containing mycotoxin (10 μg/mL), at different temperatures (21, 30, 37, 44, 50, 57, 60 °C) for different periods of incubation (15 and 30 min; 1, 2, 4, 6, 7 and 10 h). The reaction samples were diluted in the LC-MS mobile phase, filtered through PTFE syringe filters (13 mm diameter, 0.2 μm pore size), and analyzed by LC-MS. Additionally, a control assay without enzyme was prepared and subjected to the same protocol for each procedure.

### 2.4. LC-MS Method

Detection and identification of the compounds was performed using a Thermo Scientific™ Vanquish™ Flex UHPLC (Thermo Fisher Scientific, Bremen, Germany) system coupled to a Thermo Scientific™ Orbitrap Exploris™ 120 high-resolution accurate mass spectrometer (Thermo Fisher Scientific, Bremen, Germany). The instrument control and data processing were carried out by Xcalibur 4.5 software (Thermo Fisher Scientific). The UHPLC column utilized was a YMC-Triart C18 column (150 mm × 2.1 mm i.d., 3 μm particle size) protected with a guard column of the same material (2.1 mm i.d.). The mobile phases consisted of A (water with 0.1% of acetic acid) and B (acetonitrile with 0.1% of acetic acid). The flow rate was 0.35 mL/min using the following linear gradient scheme (*t* in min; % A): 0 min, 70%; 3 min, 40%; 10 min, 70%; and total run time was 15 min. The column temperature was 35 °C, the autosampler temperature was set at 15 °C, and the injection volume was 10 μL. Ion source (Thermo Scientific™ OptaMax™ NG ion source) was equipped with a heated electrospray ionization (HESI) probe. The external mass calibration of the Q-Orbitrap was performed once a week to ensure a working mass accuracy < 3 ppm. The optimized HESI temperature was set at 350 °C, the capillary temperature at 325 °C, and the electrospray voltage at 3.5 kV and 2.5 kV for positive and negative modes, respectively. Sheath and auxiliary gas were 50 and 10, respectively. All qualitative data in this study were acquired using the SIM and MS^2^ mode. In the last mode, the samples were fragmented with a normalized collision energy of 30% to obtain product ion spectra.

#### Monitoring of OTA Degradation

The enzymatic reaction was monitored by the disappearance of OTA, detected by LC-MS, and confirmed by the appearance of OTα, one of the degradation products. The identification of the peaks corresponding to OTA and OTα was validated by comparison with a standard solution and by the MS^2^ spectrum of OTA, which can be found in the Supporting Information (Figure S1). OTA quantification was performed using a calibration curve spanning the range of 0.98–500 μg/mL. The calculated limits of detection (LOD) and quantification (LOQ) were 0.8 μg/mL and 2.4 μg/mL, respectively.

### 2.5. Circular Dichroism

The secondary structure of PPL was studied by circular dichroism spectroscopy, using a Jasco J-1500 spectropolarimeter, equipped with a temperature controller set at 37 °C. The enzyme concentration was set at 5 μM, dissolved in the buffer solutions under study and in pure water. The baseline was recorded using these solvents and subtracted to the enzyme spectra. The spectra were recorded to be 190–240 nm at a scan speed of 20 nm/min and bandwidth of 1 nm. The pathlength cell was 1 mm. The final spectra were obtained by the average of three scans for each sample. The data analysis of circular dichroism was conducted using DichroWeb (http://dichroweb.cryst.bbk.ac.uk, accessed on 19 April 2024), an online tool specialized in determining the secondary structure of proteins from circular dichroism spectra.

### 2.6. Computational Studies

The model used for the computational studies was built from the X-ray crystallographic structure of PPL (PDB:1ETH; resolution: 2.8 Å) [20], which was obtained from the RCSB Protein Data Bank (https://www.rcsb.org/, accessed on 9 March 2024). Crystallization water molecules were removed, as well as 2-mercaptoethanol and (hydroxyethyloxy)tri (ethyloxy)octane molecules found in the crystallized structure. Hydrogen atoms were then added using the Open Babel software package version 2.4.1, targeting a pH of 7.

Molecular Dynamics (MD) simulations were then carried out to explore the structural relaxation of PPL under pure water and PBS. To prepare the simulation box for the structural relaxation in pure water, the structure of PPL was placed at the center of a 10.5 nm × 11.5 nm × 17.5 nm box, accompanied by 2 chloride ions for balancing the overall charge of PPL. A total of 51,700 water molecules were then added to the simulation box using the Packmol software package 20.14.3 [32]. A similar protocol was used to create the initial state of PPL in PBS, by adding 170 potassium ions, 69 monohydrogenphosphate ions and 25 dihydrogenphosphate ions. The Amber force field was used throughout the MD protocol, using the ff14SB parameters [33] for the protein structure, GLYCAM-06j [34] for the glycosylated sites of PPL, the water TIP3P model [35], and the GAFF2 [36] parameters for the remaining species, except the long-range parameters for potassium and chloride which were collected from Joung and Cheatham [37]. All calculations were carried out using the LAMMPS software [38], version dated from the 21st of November of 2023. The same MD protocol was applied to the two systems: first the strain caused by the manipulation of the PPL structure was relaxed using the hessian-free newton minimization method. After minimization, the initial velocities of the solvent atoms were created from a random distribution targeting an initial temperature of 150 K, and the solvent was allowed to heat (with the enzyme structure frozen) to 300 K in 1 ns, under NVT conditions using the Nosé–Hoover thermostat and a time step of 0.5 fs. This allowed the solvent molecules to permeate eventual vacancies in the PPL structure and prevent its collapse. The velocities of the enzyme atoms were generated targeting an initial temperature of 150 K, and the system was allowed to heat to 300 K during 5 ns. Finally, the system was equilibrated at 300 K using the NVT conditions described above for 5 ns, followed by a 20 ns production run. The statistics on the structure of PPL in each solvent were collected from the last 5 ns of this production run, the trajectory along which was sampled at a rate of 1 frame per 0.01 ns. The affinity of OTA and *p*-NPO for pure water and PBS was accessed by simulating one solute molecule in NVT conditions in a 10 nm × 10 nm × 20 nm box divided in two isolated regions along the *zz* axis, with periodic boundary conditions along the *xx* and *yy* directions. The lower part of the box was filled with pure water to the target density of 0.0033 Å^−3^, whereas the upper half was filled with a pre-equilibrated PBS solution model. The solute molecule was placed at the center of the upper half and allowed to equilibrate for 1 ns, after which a production run of 10 ns was carried out. The solute molecule was then alchemically translated to the lower half of the box (occupied by pure water), allowed to equilibrate, and subjected to a new production run.

Finally, the affinity of *p*-NPO and OTA to PPL was evaluated for the initial (crystalline) structure, as well as for the last recorded structure of PPL in both water and PBS. This was carried out using a standard docking protocol [39]. The docking studies were prepared using the MGLTools 1.5.7 software suite [40] and the binding mode search and ranking were carried out using AutoDock Vina, version 1.2.5 [41,42]. The conformational search was restricted to a box containing the putative active sites of PPL, which is comprised by the amino acid residues Ser156, Asp177, and His264 in chains A and C. A grid spacing of 1 Å was used, together with an exhaustiveness setting of 24, and collecting the 30 lowest energy binding modes within 5 kcal.mol^−1^ of the lowest energy mode.

## 3. Results and Discussion

Ultra-pure water was employed herein as the reaction medium for the enzymatic degradation of OTA by PPL. This approach seeks to refine the previously described method, enhancing its suitability for application in food or feed.

Here, the optimal conditions identified in previous studies were reproduced, and the role of water in OTA degradation by PPL was investigated, comparing the results with degradation in the presence of a buffer (phosphate buffer). Previous research has shown that PPL exhibits its highest activity in alkaline media, with a pH range from 7.3 to 9 and an optimal temperature of 35–45 °C [19]. Specifically, Milek found that at pH 7.5, the optimal temperature for olive oil hydrolysis was 36 °C [43]. Additionally, selective hydrolysis of two medicinal seed oils was successfully carried out at pH 8.0 [44]. Although PPL is an alkaline lipase, it has also been used in neutral to moderately acidic conditions (pH 6.5–7.0). Lei et al. investigated the catalytic activity of PPL in the hydrolysis of an olive oil emulsion at pH 6.9 [45]. While its optimal catalytic activity occurs in an alkaline environment, PPL demonstrates greater stability in neutral to moderately acidic media.

### 3.1. Enzymatic-Assisted Degradation of OTA in Pure Water

To elucidate the activity of PPL in ultra-pure water and phosphate buffer (100 mM, pH 7.5) for OTA degradation, an initial screening was carried out under previously established conditions at 37 °C for 10 and 24 h [13].

After 10 h in pure water, the degradation of the mycotoxin was complete, unlike what happens in phosphate buffer, where PPL degrades only 21% of OTA. After an incubation period of 24 h at the same temperature, only 40% of the mycotoxin was degraded in the buffer, compared to 100% degradation in pure water. Although the literature suggests that PPL in water is in its closed form, during OTA degradation, this enzyme appears to have activity in water, contrary to expectations.

**Figure 1:**
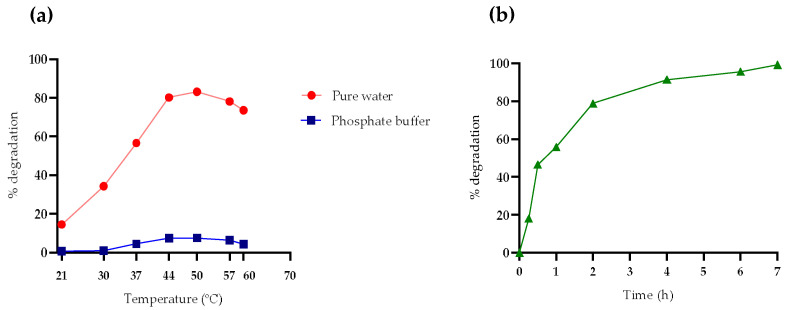
Evaluation of the optimal conditions for OTA hydrolysis by PPL in two different media. The reaction was monitored at different (**a**) temperatures (21, 30, 37, 44, 50, 57, and 60 °C) and (**b**) incubation times (15 and 30 min; 1, 2, 4, 6, 7, and 10 h). The optimal temperature for OTA hydrolysis was 44 °C in water and buffer. The incubation time required for the complete hydrolysis of OTA was 7 h in pure water (44 °C).

To evaluate the optimal temperature for PPL, tests were conducted at different temperatures over a period of 2 h in both water and buffer. The study explored temperatures above and below the reference value of 37 °C, offering a wider range of data points to identify the optimal conditions for maximizing enzymatic activity and OTA degradation efficiency. The incubation time of 2 h was chosen based on evidence showing the most significant differences in degradation percentages. The study revealed that the highest OTA degradation during this period occurred at 50 °C for both conditions, rather than at 37 °C (Figure 1a). Given the slight difference between 50 and 44 °C, the latter was preferred due to its lower energy consumption during incubation.

The experiment was reproduced at 44 °C in pure water to determine the time required for PPL to achieve complete degradation of OTA. It was observed that after 7 h, OTA was 100% degraded at 44 °C, compared to the 10 h required at 37 °C. Examining Figure 1b, rapid degradation is evident up to the second hour of hydrolysis at 44 °C, after which the process decelerates. This slowdown can be attributed to the decreasing availability of substrate as the reaction progresses or to losses in activity.

**Figure 2:**
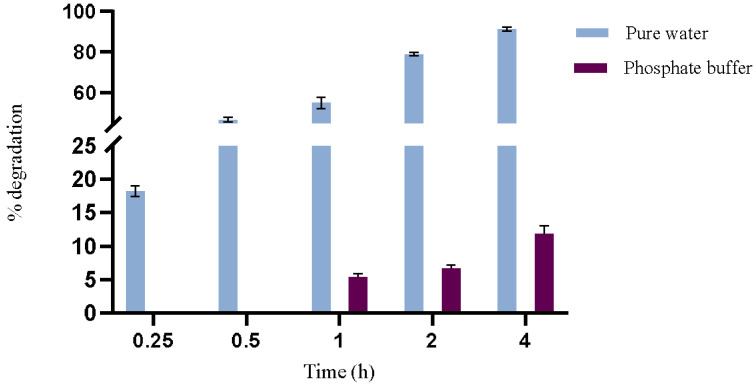
PPL enzymatic activity in pure water and in phosphate buffer (100 mM, pH 7.5) evaluated as OTA degradation for 4 h of incubation at 44 °C.

Based on preliminary optimization trials, a maximum reaction time of 4 h at 44 °C was established for the study of enzymatic activity to assess more evident differences between the reaction media. The results depicted in Figure 2 highlight a notable difference in the degradation rate of OTA when comparing pure water and phosphate buffer over different time intervals. In pure water, OTA degradation by PPL reached 18% in just 15 min and 91% in 4 h, underscoring the system’s efficiency in this solvent with a notably rapid degradation rate. Conversely, in the phosphate buffer, OTA degradation was significantly slower, with only 12% degraded after 4 h of reaction.

The study suggests that the enzyme’s stability and efficiency are influenced by the solvent. To prove this, the stability of PPL in pure water and phosphate buffer was evaluated by incubating the enzyme in these solvents at 44 °C over a time range (0.25 to 120 h). Enzyme activity was measured using the model substrate *p*-nitrophenyl octanoate, with quantification of the product *p*-nitrophenol performed using LC-MS technique.

The decay constant (K), which represents the enzyme inactivation rate, is significantly higher in the buffer (0.27) compared to water (0.14), as shown in Table 1. This increased inactivation rate suggests that, in the buffer environment, the enzyme loses its catalytic activity more rapidly. This difference is further supported by the half-life values. The half-life is shorter in the buffer (~2 h 30 min) compared to water (~4 h 4 min), indicating reduced conformational and functional stability of the enzyme in the buffer. These values were calculated using GraphPad Prism 9.0 software, allowing for precise data analysis.

These results suggest greater enzyme stability in pure water compared to phosphate buffer. The difference in stability may be attributed to ionic interactions that can destabilize proteins through conformational changes in their structure. Such structural changes can increase the enzyme’s susceptibility to denaturation or aggregation, resulting in a rapid loss of catalytic activity. Pure water, despite not providing the same buffering capacity as phosphate buffer, appears to allow for greater structural stability over a longer period.

### 3.2. The Effect of pH and Conductivity on OTA Degradation

**Figure 3:**
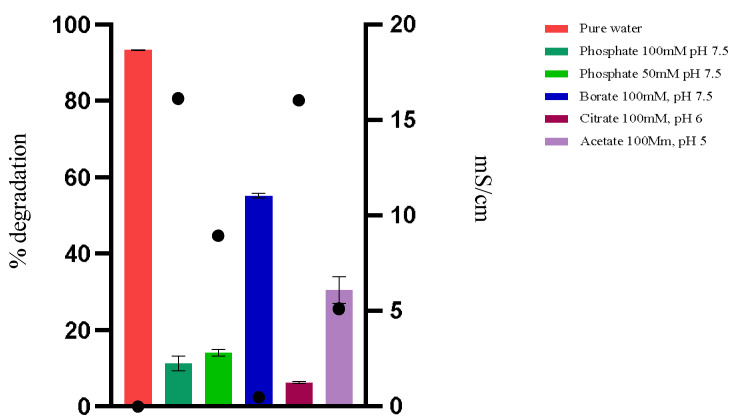
The hydrolysis of OTA by PPL in different buffers and ultra-pure water at 44 °C is represented by bars. The conductivity in mS/cm of each solution under study is represented by a black dot above each bar.

To understand the efficiency of the enzyme in water, experiments were conducted using water and different buffers, with variations in buffer type, pH, and ionic concentration, at 44 °C (Figure 3). As shown in the previous results, the enzyme appears to be more efficient in degrading OTA when in water. Ultra-pure water ideally has a pH of 7 at 25 °C, which characterizes it as neutral. However, in practice, measuring the pH of pure water can be challenging due to its low electrical conductivity and high sensitivity to environmental contamination. Small amounts of carbon dioxide from the air can dissolve in water, forming carbonic acid (H_2_CO_3_), which can slightly lower the pH to values around 5.5 to 6.5. Therefore, pure water has a slightly acidic pH and low conductivity.

The hydrolysis of OTA by PPL in phosphate buffer at pH 7.5 (100 mM) showed a much slower and less efficient performance, possibly due to the phosphate concentration. Reducing the phosphate concentration to 50 Mm did not significantly improve the degradation, indicating that the presence of phosphate, regardless of concentration, may be detrimental to the enzymatic activity (Figure 3).

The reaction in a borate buffer at pH 7.5 and 100 mM showed improvement compared to the reaction in phosphate buffer. This suggests that the type of ion in the buffer, in addition to low conductivity, significantly influences enzymatic activity. Borate may interfere less with the enzyme or substrate structure, resulting in higher activity, though it still does not match the efficiency observed in pure water. When using buffers with pH levels of 5 and 6, the enzyme’s performance at pH 5 was better than at pH 6. However, neither condition was as effective as pure water.

The results indicate that buffers, especially those with high conductivity, seem to decrease enzymatic activity, suggesting sensitivity of the enzyme to these ions and/or a change in the form of OTA that reduces its availability as a substrate. Ionic interference can compromise substrate availability for the enzyme by forming complexes between the present ions and OTA, as well as direct interactions with the enzyme structure, affecting its conformation, including the lid conformation, and consequently, its catalytic activity. Similarly, the pH can alter the ionization of OTA molecules, affecting their availability as a substrate for the enzyme. Under the pH conditions described as optimal in the literature, the ionized form of OTA may not be ideal for binding or recognition by the enzyme, thus reducing degradation efficiency.

At pH 7.5, the carboxylic group (-COOH) of OTA tends to be predominantly in the ionized form (-COO^−^), while the phenolic group (-OH) remains neutral. This ionization increases the molecule’s polarity, as deprotonated groups (forming anions) tend to be more polar. This occurs because the loss of a proton increases the electronegativity of the functional group’s atom, resulting in a more polar bond. In ultra-pure water, at a more acidic pH (~5–6), both groups may be predominantly in the protonated form, leaving the molecule with its normal less-polar character. Since water has a high dielectric constant and high polarity, the hydrophobic effect may occur, which reflects the clustering of nonpolar molecules or functional groups to reduce the polar/nonpolar interfacial area. This could lead to the concentration of OTA molecules and make them more available for reaction.

In pure water, the enzyme exhibited its best performance, indicating that low conductivity and acidic pH are ideal conditions for enzymatic action.

### 3.3. Secondary Structure of PPL—Circular Dichroism

**Figure 4:**
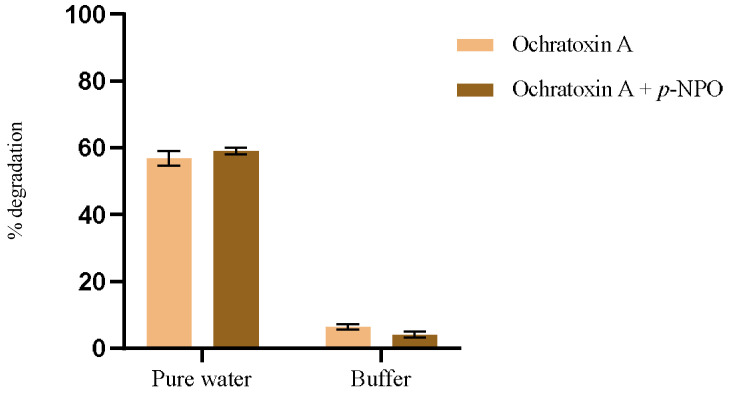
The hydrolytic behavior of PPL in water and phosphate buffer (pH 7.5, 100 mM) across different substrates was investigated.

The analysis of the secondary structure of PPL as performed by circular dichroism (CD) spectra in the various solvents studied is presented in Figure 4. Performing CD spectra in citrate buffer was not feasible due to a rapid voltage increase that would exceed the maximum recommended value. The results (Table 2) revealed significant variations in the secondary structure composition of the enzyme across different solvents. The results indicate that the enzyme exhibits a higher number of helical structures (helices 1 and 2) when in water (8.68% in total) compared to 100 mM phosphate buffer (6.8% in total). Additionally, the quantities of beta sheets (strand 1 and strand 2) are similar in both conditions, with a slight decrease in buffer (36.4% in water vs. 37.2% in buffer). The amount of turns and unordered structures also remained relatively constant between the two conditions, displaying similar values (22.1% and 32.9% in water vs. 22.7% and 33% in buffer). PPL in borate buffer exhibits a behavioral pattern like that observed in pure water (9.4% in total of helices 1 and 2). Acetate buffer showed the lowest content of alpha helices (5.6% in total), along with the highest amount of beta sheets (39.3%). These differences suggest that the aqueous environment favors the formation of helical structures in the enzyme, while some buffer solutions seem to reduce the stability of these helical structures, possibly leading to a more disordered conformation. The higher presence of alpha helices in water suggests a more stable and catalytically active conformation, resulting in a greater degradation of OTA. On the other hand, the reduction of alpha helices in some buffers indicates less favorable conformations for enzymatic activity. These results corroborate the data obtained by molecular modeling. The STRIDE [46] algorithm implemented in VMD [47], version 1.94, was used to ascertain the stability of the secondary structure during the final 5 ns of the production runs, showing a dominance of turn structures and the relative lack of helix domains, as shown in Figures S2 and S3 of the Supplementary Information.

### 3.4. Simultaneous and Isolated Hydrolysis of Ochratoxin A and a Model Substrate

Specificity is a crucial property of enzymes, encompassing both substrate and positional specificity. For PPL, natural substrates include glycerol esters and showed maximal activity on the hydrolysis of triacylglycerols between 35 and 40 °C at pH in the range from 6.7 to 7.5. The specificity of PPL in hydrolyzing various oleic acid esters, prepared with different substituents and alcohol chain lengths, was also investigated. Among several compounds tested, *p*-nitrophenyl oleate exhibited one of the highest activities, being 1.4-fold higher than triolein (the standard substrate) at alkaline pH medium [19]. The optimal conditions described in the literature for the PPL enzyme, using different substrates, differ from those we have demonstrated here as optimal for the degradation of OTA. This confirms that the ideal conditions for PPL activity are substrate dependent.

To investigate how different substrates influence enzyme efficiency, we employed the model substrate *p*-nitrophenyl octanoate as a comparative standard alongside OTA hydrolysis. This study aimed to elucidate the intricate interactions among substrate structure, reaction medium properties, and enzyme activity. Ultimately, our goal was to optimize reaction conditions for practical applications. The results for OTA degradation in both solvents, in the presence and absence of the model substrate, are represented in Figure 4. The conditions for evaluating OTA degradation were like those performed previously, at 44 °C for 1 h. The hydrolysis reaction of *p*-NPO was conducted following the procedure for activity measurement.

The hydrolytic behavior of PPL exhibits distinct differences between water and phosphate buffer (pH 7.5, 100 mM) across different substrates. Specifically, for OTA, degradation in pure water is observed to be 15 times higher compared to that in phosphate buffer (Figure 4). Conversely, with the model substrate, rapid hydrolysis occurs in the phosphate buffer, whereas it proceeds much slower in water.

The possibility of ionization affecting substrate arrangement and subsequent degradation is relevant in explaining the differences observed between OTA and *p*-NPO.

In water, OTA is in a monoanionic form, with the carboxylic group fully deprotonated. The presence of these negative charges generally increases the solubility of OTA in water, as ionic interactions favor dispersion in the aqueous medium. In contrast, *p*-NPO maintains the phenolic group in its protonated form, resulting in a neutral molecule. The neutrality of *p*-NPO tends to reduce its solubility in water, making it less soluble compared to OTA.

When conducting the competitive assay with both substrates present in the same solution, the results remain consistent, showing higher degradation of the model compound in buffer and OTA in water. This suggests that the interactions between the enzyme, OTA, and the model substrate are independent of each other and do not interfere with one another.

These results highlight the complexity of interactions between enzymes, substrates, and reaction medium. The influence of the reaction medium may vary depending on the specific properties of each substance and the specific interactions that occur during the reaction.

### 3.5. Computational Insights on the Structure of PPL in Water and PBS

**Figure 5:**
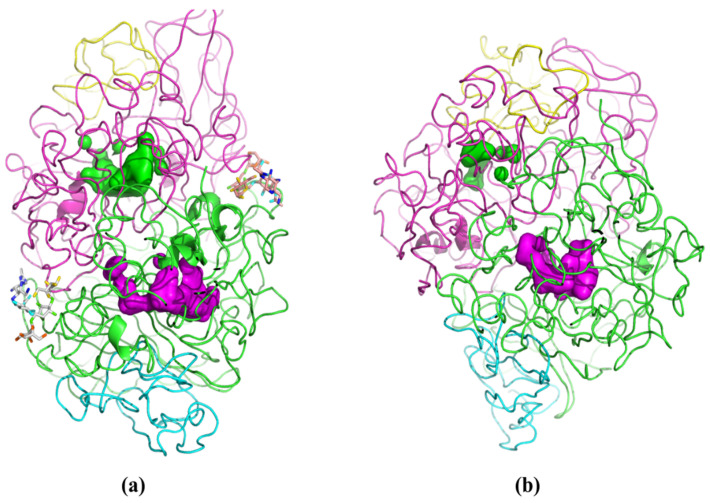
Cartoon representation of the last recorded conformation of PPL in water (**a**) and PBS (**b**), the cavities of the active centers are highlighted for chain A (magenta surface) and chain C (green surface). Both images are cropped depth-wise to aid visualization of the active center cavities.

Molecular modeling techniques were employed to rationalize the comparative performance of PPL on the hydrolysis of OTA in water and phosphate buffer (PBS). These studies were partially hindered by the relatively low quality score of the 1ETH structure in the PDB register. Indeed, the PPL moiety was noticeably different from the crystallographic structure at the end of the energy minimization procedure. Despite this, the PPL structure stabilized during the NVT MD simulations, achieving a Root Mean Square Displacement (RMSD) of the protein backbone of 16.8 ± 0.8 Å in water, and 15.2 ± 0.7 Å in PBS, using the crystallographic structure as reference. When analyzing the enzyme’s backbone, it was observed that in water, the enzyme shows greater movement compared to PBS. Moreover, the results from the MD simulations suggest that the structural relaxation of PPL in the two media took place in a divergent manner, with an RMSD of 10.9 Å between the final structures recorded in medium. Visual inspection of the structure of PPL in either media (Figure 5) highlights the preservation of both active center cavities in water (Figure 5a). In PBS medium, the cavity corresponding to the active center of subunit C (represented by the green volume inside the magenta chain in Figure 5b) is strangled, which may hinder the ability of the substrate to bind to the active site in PBS.

**Figure 6:**
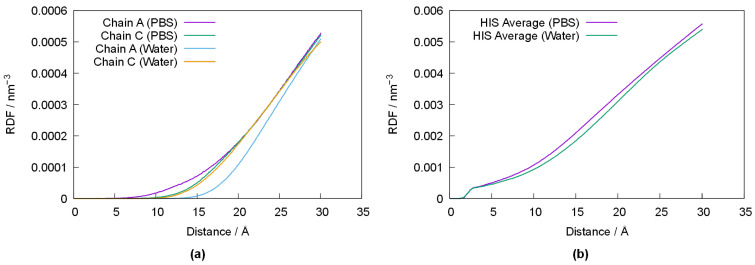
Radial-pair distribution function of the oxygen atom of the water molecules (Ow) with respect to the average position of the active centers of PPL’s chains A and C in water and PBS (**a**). RDF of Ow with respect to the average position of the histidine residues in PPL in water and PBS (**b**).

In order to better ascertain the impact of the varying conformation of PPL with respect to its media, Figure 6 depicts the radial-pair distribution function (RDF) of water’s oxygen atom (OW) relative to the average position of Ser156, Asp177 and His264 in chains A and C of PPL in both media (Figure 6a), as well as the RDF of OW relative to the average position of all histidines in chains A and C (Figure 6b). Despite some differences between chain A and C (which are likely the result of error propagation from small differences in the original crystallographic structure), the results depicted in Figure 6a show that the active centers are somewhat closer to the water–protein interface in PBS, and that solvation of PPL in pure water results in the active centers being pushed towards the inside of the enzyme. Indeed, this behavior is observed for all histidine residues, as shown in Figure 6b, suggesting a migration of the histidine residues towards the inner (more lipophilic) moiety of PPL when in water.

OTA is a molecule with intermediate polarity, so the more exposed location of the active center of PPL in PBS may lead to competition within the cavity between the hydrolysis reaction and the solvation of OTA. However, the more internal location of the active center in chain A in water may help to keep OTA close to the active center, increasing the likelihood of forming a more effective enzyme–substrate complex.

When we assess the degradation of the model compound, we postulate that this subtle effect may justify the observed results, based on the different lipophilic character of *p*-NPO and OTA. Indeed, *p*-NPO, being less polar than OTA, may suffer from some salting-out-like effect in PBS, and adsorb to the surface of PPL. This would imply different binding energies of *p*-NPO to the two PPL conformations. Confirmation of this hypothesis was carried out using molecular docking studies of *p*-NPO and OTA with PPL at the last recorded conformation in water and PBS, as well as in the crystallographic conformation, for reference.

The binding energies of the compounds *p*-NPO and OTA to the PPL enzyme were evaluated under three conditions: in the crystallographic structure, in PBS buffer, and in water, considering the lowest energy binding mode (L) and the smallest distance to the active center (C). The results of the docking studies are summarized in Table 3, showing that the relaxation of the PPL structure has a detrimental effect on the binding ability of both OTA and *p*-NPO to PPL. Moreover, the lowest binding mode to the crystallographic structure of PPL is also a binding mode to one of its active centers, with the carbonyl of the ester (in *p*-NPO) or amide (in OTA) groups oriented towards His264. On the other hand, the lowest binding mode of either OTA and *p*-NPO to the solvated forms of PPL places the substrate away from the active center, prompting a manual search for the lower energy binding mode that presents the substrate close enough to the active center and with the correct orientation.

As shown in Table 3, the binding energies are higher for both substrates in water. In the case of *p*-NPO in PBS, such mode is quasi degenerate with the lowest energy one, whereas in water, this node lies considerably higher in energy. These results suggest that adsorption to an inactive moiety of the enzyme may be responsible for PPL’s relatively poor performance in degrading *p*-NPO in water. In the case of OTA, the closest-to-active center mode in PBS is only slightly above the lowest energy binding mode (by about 1 kcal/mol), suggesting that the interaction between substrate and active center is achievable in both water and PBS. Thus, the docking results suggest that the increased activity of PPL toward the degradation of OTA in water is a combination of two factors: from the OTA-PPL interaction, the binding energy suggests greater affinity between enzyme and substrate in water, relative to PBS; from the OTA/*p*-NPO competition, the results suggest that, in water, much of *p*-NPO will bind to PPL in an inefficient manner, while PPL’s active sites remain available for OTA to bind in an energetically favorable manner. Moreover, the simulations of OTA and 4NPO in pure water and PBS show that Ota has greater affinity towards PBS (Δ_W→PBS_ E = −52 kcal/mol), whereas *p*-NPO shows a slight preference for pure water (Δ_W→PBS_ E = +7 kcal/mol). These results further suggest that a “salting out” effect could be driving 4NPO into PPL when in PBS buffer, while OTA would tend to remain in the PBS medium.

## 4. Conclusions

This study shows that ultra-pure water is an effective medium for OTA degradation by PPL, achieving 91% degradation in 4 h at 44 °C, significantly outperforming phosphate buffer, which reached only 12% in 4 h. The enhanced efficiency in water is attributed to its low ionic interference, which improves PPL stability (half-life of 4 h compared to 2.5 h in phosphate buffer) and maintains enzyme conformation conducive to OTA degradation. Molecular modeling confirmed that water increases enzyme mobility and OTA binding affinity, resulting in higher activity compared to buffers.

This study positions ultra-pure water as a green, safe, and efficient solvent for mycotoxin degradation, with potential applications in food decontamination. Future research should focus on the decontamination of agriculture matrices and/or immobilization of PPL for enhanced reusability and broader industrial applications.

## Figures and Tables

**Table 1:** Half-life decay parameters for PPL enzymatic activity in ultra-pure water and phosphate buffer (100 mM, pH 7.5), calculated using GraphPad Prism.

	Ultra-Pure Water	Phosphate Buffer
Decay constant (K)	0.14 ± 0.06	0.27 ± 0.11
Half-life (h)	4.74 ± 1.8	2.56 ± 0.98
Initial activity (Y_0_)	1.02 ± 0.09	1.20 ± 0.12
R^2^	0.90	0.87

**Table 2:** The analysis of circular dichroism (CD) spectra in the various solvents studied was conducted using DichroWeb software (http://dichroweb.cryst.bbk.ac.uk, accessed on 19 April 2024).

	Helix 1	Helix 2	Strand 1	Strand 2	Turns	Unordered
Pure water	0.8	7.8	24.2	12.2	22.1	32.9
Phosphate buffer 100 mM	0	6.8	24.4	12.8	22.7	33.3
Phosphate buffer 50 mM	0	6.8	24.4	12.8	22.7	33.3
Borate buffer 50 mM	1.4	8	23.2	12.1	22.3	33
Acetate buffer 50 mM	0	5.6	26	13.3	22.1	33

**Table 3:** Binding energies (in kcal/mol) of the lowest energy binding (L) mode of *p*-NPO and OTA to PPL, and of the mode showing the smallest distance to the active center (C) of PPL in the crystallographic conformation, as well as in the last recorded conformation recorded during the MD simulations in water and PBS.

Compound	PPL (Cryst.)	PPL (PBS)	PPL (Water)
*p*-NPO (L)	−7.3	−4.7	−7.6
*p*-NPO (C)	−7.3	−4.1	−5.4
OTA (L)	−10.8	−7.6	−8.1
OTA (C)	−10.8	−6.8	−7.1

## Data Availability

The original contributions presented in this study are included in the article/Supplementary Material. Further inquiries can be directed to the corresponding author.

## Supplementary Materials

The following supporting information can be downloaded at: https://www.mdpi.com/article/10.3390/foods14030397/s1, Figure S1: Chromatogram (a) and MS^2^ spectrum (b) of ochratoxin A using a Thermo Scientific™ Vanquish™ Flex UHPLC system coupled to a Thermo Scientific™ Orbitrap Exploris™ 120 high-resolution accurate mass spectrometer (Thermo Fisher Scientific, Bremen, Germany); Figure S2: Time evolution of the chain a (a), b (b), c (c), and d (d) in secondary structure of PPL is determined by the STRIDE algorithm during the final 5 ns of the production run in PBS medium; Figure S3: Time evolution of the chain a (a), b (b), c (c), and d (d) in secondary structure of PPL determined by the STRIDE algorithm during the final 5 ns of the production run in pure water.
