# Supplementary material for: Enzymatic Degradation of Ochratoxin A: The Role of Ultra-Pure Water
Source: Foods. 2025 Jan 25;14(3):397. doi: 10.3390/foods14030397 (PMC11817770; doi:10.3390/foods14030397)
Supplement: Supplementary file 1 [file foods-14-00397-s001.zip › foods-3395912-supplementary.pdf]

Supplementary Information

Enzymatic Degradation of Ochratoxin A: The Role of Ultra-Pure Water

(a)

(b)

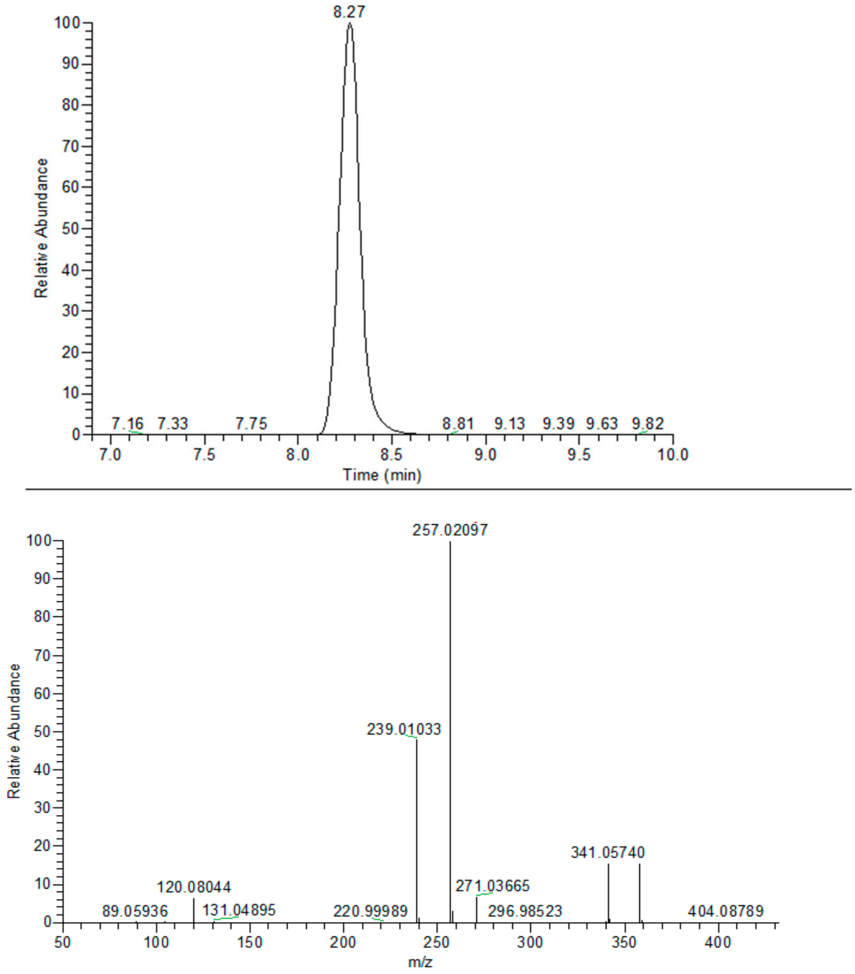

Figure S1. Chromatogram (a) and MS^2 spectrum (b) of ochratoxin A using a Thermo Scientific™ Vanquish™ Flex UHPLC system coupled to a Thermo Scientific™ Orbitrap Exploris™ 120 high resolution accurate mass spectrometer (Thermo Fisher Scientific, Bremen, Germany).

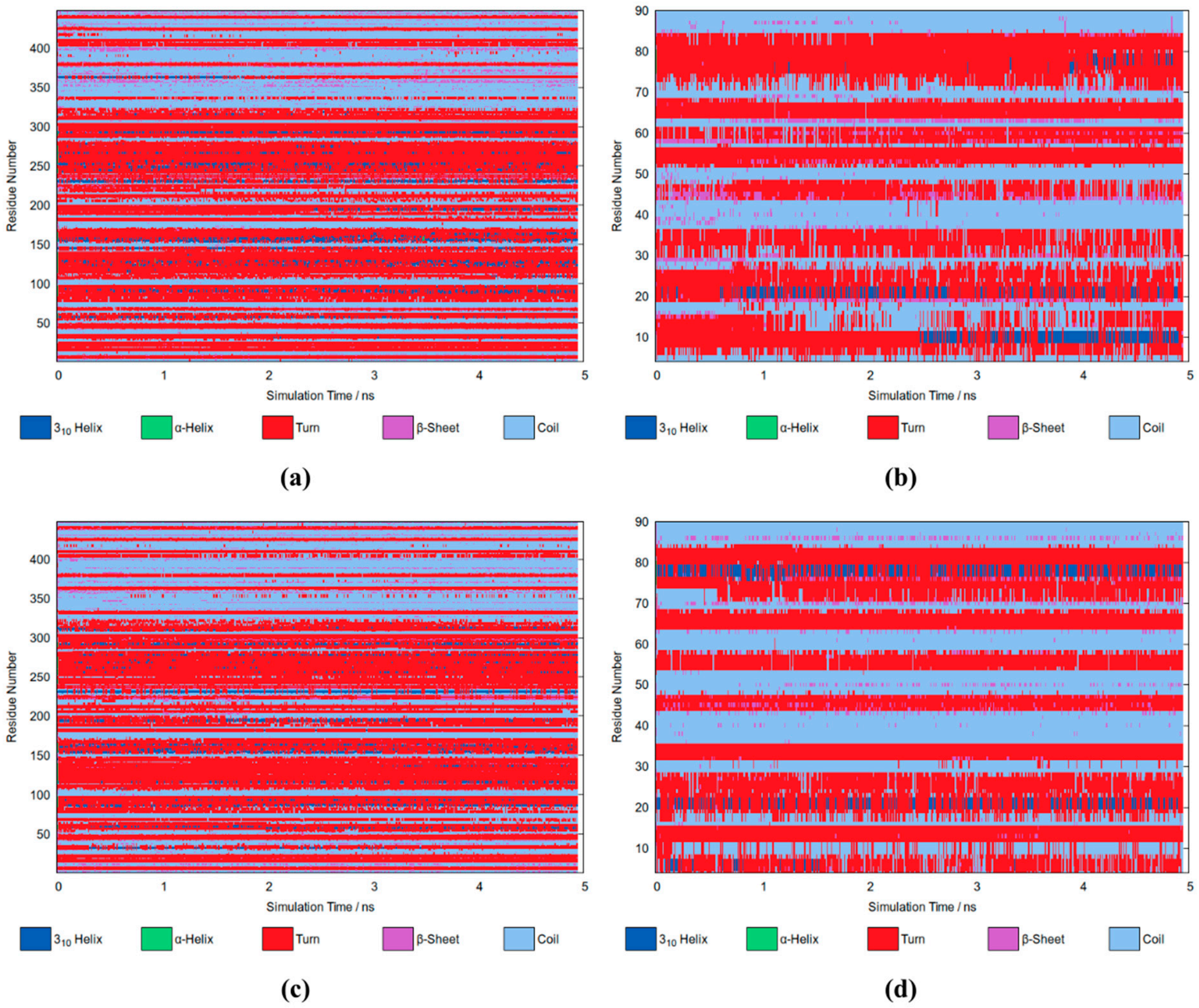

Figure S2. Time evolution of the chain a (a), b (b), c (c) and d (d) in secondary structure of PPL is determined by the STRIDE algorithm during the final 5 ns of the production run in PBS medium.

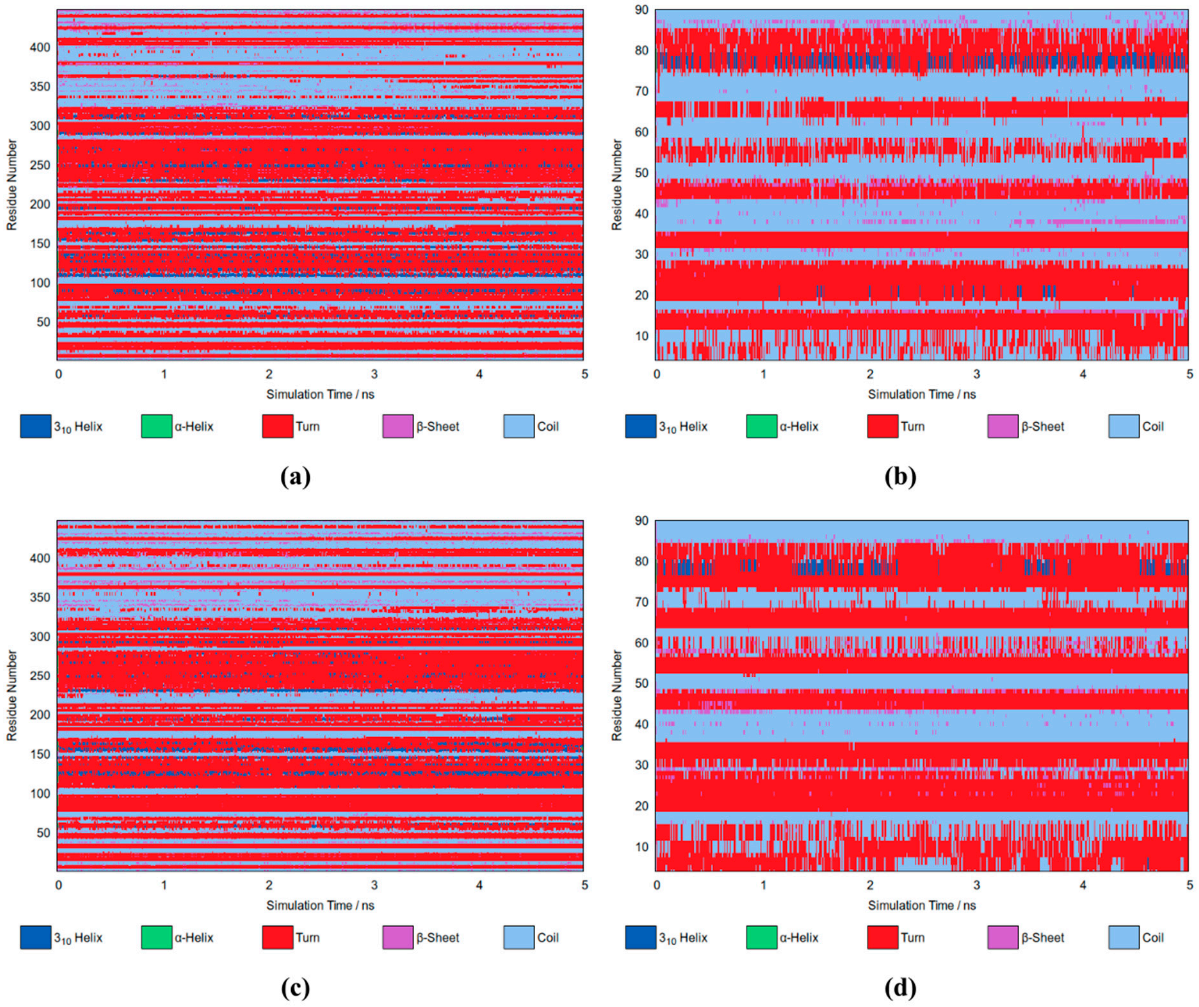

Figure S3. Time evolution of the chain a (a), b (b), c (c) and d (d) in secondary structure of PPL determined by the STRIDE algorithm during the final 5 ns of the production run in pure water.
